# Characteristics and outcomes of patients with advanced non-small-cell lung cancer who declined to participate in randomised clinical chemotherapy trials

**DOI:** 10.1038/sj.bjc.6604982

**Published:** 2009-03-17

**Authors:** C Tanai, H Nokihara, S Yamamoto, H Kunitoh, N Yamamoto, I Sekine, Y Ohe, T Tamura

**Affiliations:** 1 Department of Medical Oncology, National Cancer Center Hospital, Tokyo, Japan; 2 Cancer Information Services and Surveillance Division, Center for Cancer Control and Information Services, National Cancer Center, Tokyo, Japan

**Keywords:** randomised clinical trial, trial participation, trial effect, lung cancer

## Abstract

There are inadequate data on the outcomes of patients who declined to participate in randomised clinical trials as compared with those of participants. We retrospectively reviewed the patient characteristics and treatment outcomes of both participants and non-participants in the two randomised trials for chemotherapy-naive advanced non-small-cell lung cancer. Trial 1 compared four platinum-based combination regimens. Trial 2 compared two sequences of carboplatin plus paclitaxel and gefitinib therapies. Nineteen of 119 (16%) and 153 (37%) patients declined to participate in Trials 1 and 2, respectively. Among the background patient characteristics, the only variable associated with trial participation or declining was the patients' attending physicians (*P*<0.001). Important differences were not observed in the clinical outcomes between participants and non-participants, for whom the response rates were 30.6 *vs* 34.2% and the median survival times were 489 *vs* 461 days, respectively. The hazard ratio for overall survival, adjusted for other confounding variables, was 0.965 (95% confidence interval: 0.73–1.28). In conclusion, there was no evidence to suggest any difference in the characteristics and clinical outcomes between participants and non-participants. Trial designs and the doctor–patient relationship may have an impact on the patient accrual to randomised trials.

Randomised clinical trials (RCTs) are the definitive method for comparing the efficacy of treatments and a crucial step in the development of new cancer treatments. There has always been a big problem that their low accrual rates limit their progress (Lara *et al*, 2001; Corrie *et al*, 2003; Go *et al*, 2006).

A number of studies have examined the motivations of patients for accepting or declining entry to RCTs (Jenkins and Fallowfield, 2000; Madsen *et al*, 2000, 2002; Ellis *et al*, 2001; Wright *et al*, 2004; Ho *et al*, 2006; Albrecht *et al*, 2008). The results of questionnaire surveys administered to patients regarding clinical trials revealed that two of the most common reasons for entering the trial were the hope for personal benefit and the opportunity to contribute to the research knowledge thereby benefiting others in the future (Jenkins and Fallowfield, 2000; Madsen *et al*, 2000, 2002; Ellis *et al*, 2001; Wright *et al*, 2004; Albrecht *et al*, 2008). On the other hand, the common reasons for declining participation were worries about the process of randomisation, overestimation of the benefits of standard therapy and fear of the trial's experimental nature (Jenkins and Fallowfield, 2000; Ellis *et al*, 2001; Ho *et al*, 2006).

However, inadequate data are available on the actual outcomes of non-participants compared with those participating in RCTs (Schmoor *et al*, 1996; Braunholtz *et al*, 2001; Burgers *et al*, 2002; Peppercorn *et al*, 2004; West *et al*, 2005). Although several reports and their review (Braunholtz *et al*, 2001) have suggested the existence of a ‘trial effect’, in which participants enjoy favourable outcomes, others, especially those which attempted to exclude the confounding factors, have refuted this finding (Schmoor *et al*, 1996; Burgers *et al*, 2002; Peppercorn *et al*, 2004; West *et al*, 2005).

On the other hand, if participation in prospective trials is associated with certain clinical characteristics of the patients, generalisability of the conclusion from the data to the clinical practise, even in patients who meet the restrictive eligibility criteria, should be in question.

The purpose of this study was to analyse the characteristics and outcomes of the patients who met the eligibility criteria but declined to participate in RCTs, as compared with those who did participate, and to search for clues to improve patient accrual to clinical trials.

## Materials and methods

Between October 2000 and October 2005, each of the 272 patients, who fulfilled the entry criteria of our top priority studies during the period, was informed of all aspects of RCTs on non-small-cell lung cancer (NSCLC) and was invited to participate in one of the two trials to be conducted at the National Cancer Center Hospital, Tokyo, Japan. We make it a rule for each patient with advanced lung cancer to be hospitalised for the first-line chemotherapy. All patients are then checked for the eligibility criteria of clinical trials available at the time and recorded in our database, whether or not they are treated on trials.

Signed informed consent was obtained from the patients for future statistical analysis of their clinical courses and outcomes, even when they were treated outside clinical trials.

Trial 1 was conducted to compare the four platinum-based combination regimens (cisplatin–irinotecan, carboplatin–paclitaxel, cisplatin–gemcitabine and cisplatin–vinorelbine) in patients with untreated advanced NSCLC between October 2000 and June 2002 (Ohe *et al*, 2007). When patients declined to participate, cisplatin-based combination regimens, such as cisplatin–irinotecan, the reference arm of the trial, were recommended. The patients ultimately selected the treatment following discussions with their families and the physicians.

Trial 2 was conducted between June 2003 and October 2005 to compare the following two treatment arms; (A) four courses of carboplatin and paclitaxel (CP) followed by gefitinib, and (B) gefitinib until disease progression followed by CP, in patients with advanced NSCLC (Nokihara *et al*, 2008). When patients declined to participate, platinum-based combination regimens, such as CP, were recommended. The patients ultimately selected the treatment following discussions with their families and the physicians; treatment options included gefitinib as first-line chemotherapy, when the patients and their families wished to start with it.

Patients in each trial had to meet the following criteria: histologically and/or cytologically documented NSCLC; clinical stage IV or IIIB (including only patients with no indications for curative radiotherapy); no earlier systematic chemotherapy; at least one measurable lesion; age 20–74 years old; Eastern Cooperative Oncology Group Performance Status (PS) of 0 or 1; adequate haematological, hepatic and renal functions; and partial pressure of arterial oxygen of 60 torr or more. Each patient was required to submit a written informed consent before entry.

Four physicians (A, B, C and D) participated in Trial 1 and five physicians (A, B, C, D and E) in Trial 2. All were male. Physicians A, B, C and D had 16, 14, 11 and 9 years of experience, respectively, at the time of activation of Trial 1 (October 2000), and Physician E had 9 years of experience at the start of Trial 2 (June 2003). One of the five attending staff physicians and one to two residents or trainees attended each consultation. Which doctor actually offered the RCTs depended on each case and was not recorded, but the attending staff physician finally confirmed the decision by the patient.

Paper and/or electronic medical records from the initial visit to our centre to the end of the follow-up were retrospectively reviewed. Demographic data (age, gender, smoking history), medical information (tumour histology, clinical stage, performance status, therapy characteristics), and clinical outcomes (response rate, follow-up time, overall survival time, 1- and 2-year survival rates) were abstracted and analysed. The response was evaluated according to the Response Evaluation Criteria in Solid Tumours (RECIST) (Therasse *et al*, 2000) by the attending physicians. It is our policy to assess clinical responses with RECIST, even in routine practise. Follow-up time at our institution was defined as the period from the initiation of the first day of the initial therapy or decision of no therapy, to the last day at our institution (including death during follow-up). Survival data of the patients who left our institution could be collected by enquiry into official agency for family registry in Japan.

*χ*^2^-tests and logistic regression analysis was used to assess associations between patient characteristics and the rate of declining to participate. Overall survival (OS) curves were produced using the Kaplan–Meier method and compared with the log rank test. All participants (those who agreed to be enroled into the RCT) and non-participants (those who declined to participate in the RCT) were included in the OS analysis. A Cox proportional hazards model was used to adjust for other potential confounding factors (age, gender, smoking history, clinical stage and PS) in comparing the OS of participants and non-participants. *P*-values <0.05 were considered statistically significant. The data collected were analysed using an SPSS II statistical package.

Japanese ethics guidelines for clinical and epidemiological studies, which took effect in August 2007, do not mandate institutional review board (IRB) approval for a single-institutional, retrospective data analysis from the medical charts, when the pre-designated person of the institution so judges. This study was thus exempted from ethical review of IRB in due process, on the judgment of the responsible official, deputy director of National Cancer Center Hospital.

## Results

There were no significant differences in the outcomes between the arms of each trial. In Trial 1, no statistically significant differences in the response rate, progression-free survival and OS were observed between the four regimens. In Trial 2, there were no statistically significant differences in the median survival time (MST) (18.8 and 17.2 months) and the survival rate at 1 year between the two arms. Seventy-five patients declined to participate in those trials, and 1 of the 197 who initially accepted entry withdrew consent, refusing to continue the trial immediately after randomisation.

Table 1 shows the patient characteristics and rate of declining. 100 patients accepted and 19 patients (16%) declined entry to Trial 1, and 96 patients accepted and 57 patients (37%) declined entry to clinical Trial 2 (including the one patient already mentioned who withdrew consent after randomisation) (*P*<0.001). No significant influence on the rate of declining of patient gender, age, smoking history, tumour histology, clinical stage or PS was observed (Table 2). There were, however, large differences in the rates of decline among the attending physicians who informed the patients about the trials and asked them to participate (*P*<0.001).

The treatment regimens for those who declined participation in the clinical trials were as follows. The majority of those who declined participation in Trial 1 selected one of the four platinum-based combination regimens presented in the trial: cisplatin–irinotecan 4, cisplatin–vinorelbine 3, cisplatin–gemcitabine 1, carboplatin–paclitaxel 4. Three patients in Trial 1 desired to have no more active treatments and opted for supportive care only, but later received active treatment at their referred hospitals. The detail of their therapy is unknown.

The majority of those who declined participation in Trial 2 selected carboplatin-based combination chemotherapy: carboplatin–paclitaxel 34 and carboplatin–gemcitabine 11, there by reflecting the shift to carboplatin for advanced NSCLC in Japan at the time of Trial 2, on the basis of the reports on the activity of the carboplatin-based regimens (Kelly *et al*, 2001; Schiller *et al*, 2002; Ohe *et al*, 2007). Twelve patients (21%) selected gefitinib as first-line chemotherapy.

Survival was analysed for all of the 196 participants and 76 of the non-participants. Post-therapy was analysed for all of the 196 participants and 73 of the non-participants, who were treated at our centre. There was one possible treatment-related death due to perforation of the colon during gefitinib treatment in Trial 2. No other toxic deaths were observed among either participants or non-participants. More participants of both the clinical trials were given four cycles or more of the first-line chemotherapy, probably reflecting protocol regulations (Table 3).

Table 4 summarises the treatment after the initial therapy. There were no significant differences between participants and non-participants in the number of chemotherapy regimens. Six (8%) of those who declined participation in the trial later participated in early-phase clinical trials of experimental therapies.

We have observed no clinically relevant differences in the clinical outcomes between participants and non-participants (Table 5). Clinical response to the initial therapy was analysed for all of the 196 participants and 73 of the non-participants, excluding three patients who were not treated at our institute. The response rate was 30.6% in participants and 34.2% in non-participants (*P*=0.325). The median follow-up time at our centre was 388 days for participants and 406 days for non-participants, which was not statistically different.

**Figure 1:**
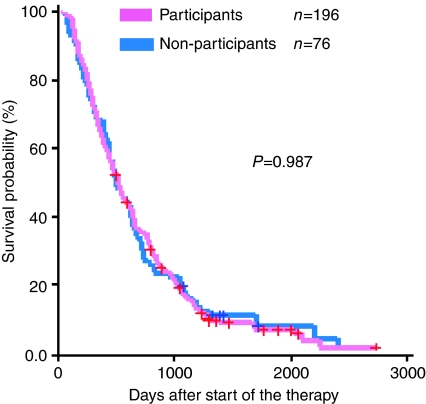
Overall survival of those who declined to participate in randomised trials (blue line, *n*=76) as compared with the participants (pink line, *n*=196). No significant difference can be observed.

**Figure 2:**
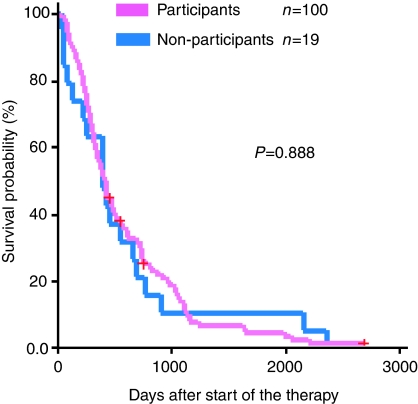
Overall survival of those who declined to participate in Trial 1 (blue line, *n*=19) as compared with the participants (pink line, *n*=100). No significant difference can be observed.

**Figure 3:**
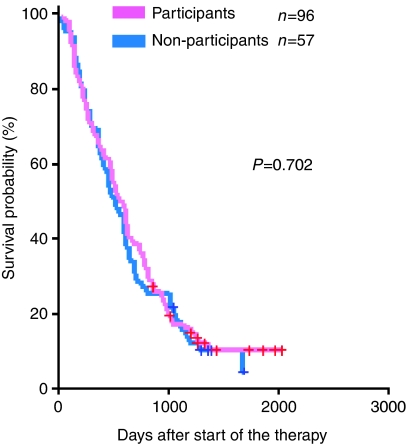
Overall survival of those who declined to participate in Trial 2 (blue line, *n*=57) as compared with the participants (pink line, *n*=96). No significant difference can be observed.

The OS was not different between participants and non-participants (Table 5 and Figure 1), with a hazard ratio of participants *vs* non-participants of 0.998 (95% confidence interval: 0.76–1.32). No significant difference in OS was observed either in Trial 1 (Figure 2) or in Trial 2 (Figure 3).

With the Cox proportional hazards model adjusted for gender, age, smoking history, clinical stage and PS, the hazard ratio of participants *vs* non-participants was 0.965 (95% confidence interval: 0.73–1.28, *P*=0.805). Among the patient characteristics, PS was the only significant factor associated with OS in multivariate analysis (*P*=0.006, by Cox proportional model).

## Discussion

It has been argued that trial participants have better outcomes than those who are not enroled in clinical trials. Several investigations have reported a favourable overall trend with trial entry (Braunholtz *et al*, 2001; Peppercorn *et al*, 2004; West *et al*, 2005). This ‘trial effect’ could derive from several factors, such as protocol effect (the way treatments are delivered), care effect (extra care related to data gathering), Hawthorne effect (changes in doctor or patient behaviour on the basis of the knowledge that they are under observation) or placebo effect (psychologically mediated benefits) (Braunholtz *et al*, 2001; Peppercorn *et al*, 2004).

In majority of the reports comparing outcomes between participants and non-participants of clinical trials, however, the non-participant ‘controls’ were chosen from differently pooled database, which could include baseline imbalances between groups and hindsight bias (Davis *et al*, 1985; Braunholtz *et al*, 2001; Peppercorn *et al*, 2004). In this study, we compared the characteristics and outcomes of those who met the eligibility criteria but declined to participate in randomised trials, and instead chose to receive standard therapy. We thus aimed at excluding confounding factors as much as possible.

On the other hand, physician triage is pointed out to be one of the barriers to cancer clinical trial accrual (Lara *et al*, 2001; Corrie *et al*, 2003; Go *et al*, 2006; Ho *et al*, 2006). We excluded the barrier by making it a rule to offer clinical trials to every patient with advanced NSCLC who satisfied the eligibility criteria.

The response rate, MST, 1-year and 2-year survival rates were all similar in both groups. We have to admit that response evaluation might not be as strict in off-protocol therapy. However, the hazard ratio for the OS was very close to 1. Although the confidence interval of 0.73 to 1.28 could not rule out the existence of clinically important difference in the treatment effect, it could not by any means be taken as a clinically relevant prognostic factor. We thus believe this confidence interval of the adjusted hazard ratio, 0.73–1.28, was narrow enough to justify the conclusion that the clinical outcomes of trial participants and non-participants were not different in our study. The differences in the number of cycles of chemotherapy given to participants and non-participants may suggest the so-called protocol effect (Braunholtz *et al*, 2001; Peppercorn *et al*, 2004), in which explicit careful description of treatment regimens could lead to improvement of outcomes. On the other hand, there clearly existed no ‘care effect’ representing the differences in incidental aspects of treatment or care between participants and non-participants, which the protocol may require, such as extra follow-up or extra nursing care (Braunholtz *et al*, 2001; Peppercorn *et al*, 2004). In our cases, the same treatment teams took charge of and followed both groups of patients in the same manner, and found no differences in the post-treatment characteristics or follow-up periods. Thus, our first finding was that the clinical trials themselves seemed to have no influence on the outcomes or pattern of care of the patients.

The second finding was that we could not find any demographic characteristics to influence the patients' willingness to participate in clinical trials. Taken together with the first finding, both the characteristics and outcomes of the non-participants were very similar to the participants. This would imply that the participants ably represented the whole patient population of the disease status who met the eligibility criteria, and that conclusions from the clinical trials could be generalised.

Our study, however, could only show the similarity in the prognosis of the participants and non-participants, and, unlike an earlier report (Link *et al*, 1986), not that of the treatment effect itself. This could not be evaluated because there were no significant differences in the clinical effect between the arms in both Trial 1 and Trial 2. If newer, much more effective experimental treatment were presented in the trials, the outcome could be better in trial participants, which was the case in the adjuvant chemotherapy trial for osteosarcoma (Link *et al*, 1986). In that report, eligible patients who declined randomisation, but were given adjuvant chemotherapy, also had better outcomes. Therefore, a very effective treatment could lead to a better outcome both on and off trial. Ideally, strict comparison of the effects of the study participation itself would require randomised design of the trial participation (Braunholtz *et al*, 2001; Peppercorn *et al*, 2004), which is almost impossible to conduct.

Thirdly, the declining rate seemed to be influenced by the trial design. Trial 1 was the comparison of four similar platinum-doublet regimens. On the other hand, Trial 2 was the comparison of two arms with sequentially different types of chemotherapy. In general, people might have the impression that injection therapy would be more effective, and less convenient, than oral administration. It is easy to understand that more patients felt difficulty in accepting the randomisation of different types of therapy, such as Trial 2 (Schmoor *et al*, 1996; Jenkins and Fallowfield, 2000).

The declining rate also seemed to be greatly affected by the attending physician. The attending physician with longer experience as a thoracic oncologist tended to have lower rate of declination. Even though we do not have records on who actually informed the participants regarding the trial, residents or trainees under Physician A seemed to have had more chance to lead the consultation, which might have affected the rate of declination. Trust in the doctor is one of the most important reasons for agreeing to enter an RCT, whereas it has also been cited as the main reason for declining to participate (Jenkins and Fallowfield, 2000; Ellis *et al*, 2001; Stryker *et al*, 2006). Patients prefer the doctor to make the treatment decisions rather than to be randomised. A recent report emphasises the influence of physicians' clinical communication on patients' decision-making on participation in clinical trials (Albrecht *et al*, 2008). Improving communication and more interventions by clinical research coordinators and other medical staff members in all eligible patients may improve the accrual rate (Fallowfield *et al*, 1998; Wright *et al*, 2004; Stryker *et al*, 2006).

Finally, it was interesting to find that 8% of those who declined the RCTs participated in early-phase trials during follow-up. It is possible that the lack of effective therapies had changed their recognition of clinical trials. However, it might support the psychological states of patients as reported in earlier studies (Jenkins and Fallowfield, 2000; Ellis *et al*, 2001; Wright *et al*, 2004); patients expect experimental therapies to give them improved effectiveness but with fear of uncertainty. They are reported to have negative opinions regarding the principle of randomisation. Better understanding of the patients' decision-making process and the factors influencing their psychological states may lead to improvement in RCT accrual.

Our study has several limitations. One is that it was conducted at a single academic institution; the situation might well have been different in others or when the research was performed on a multi-institution basis. The second is that we analysed data from only two trials and could not definitely conclude that a trial design would affect the patient accrual. Third, we have no data on the reasons for patient participation. That information would be definitely useful for analysing factors for consent or declining to participate, and would help to improve the accrual rate. Further research is required.

In conclusion, there was no evidence of any difference in the response rates and survival times between participants and non-participants. The declining rate of clinical trials was influenced by the referring physicians and trial designs. Further analysis of the decision-making process of those offered trials is warranted, for it may improve patient accrual to RCTs.

## Figures and Tables

**Table 1:** Patient characteristics and rate of declining

	**Clinical trial 1**			**Clinical trial 2**			**Total**		
	**P**	**NP**	**ROD (%)**	**P**	**NP**	**ROD (%)**	**P**	**NP**	**ROD (%)**
No.	100	19	16	96	57	37	196	76	28
*Gender*									
Male	64	12	16	55	34	38	119	46	28
Female	36	7	16	41	23	36	77	30	28
									
*Age*									
<60	46	9	16	37	29	44	83	38	31
⩾60	54	10	16	59	28	32	113	38	25
									
*Smoking history*									
+	69	9	12	55	33	38	124	43	26
−	31	10	24	41	24	37	72	33	31
									
*Clinical stage*									
III	24	6	20	21	19	48	45	25	36
IV	76	13	15	75	38	34	151	51	25
									
*PS*									
0	27	4	13	47	19	29	74	23	24
1	73	15	17	49	38	44	122	53	30
									
*Physicians*									
A	32	5	14	23	25	52	55	30	35
B	28	0	0	25	1	4	53	1	2
C	18	2	10	34	4	11	52	6	10
D	22	12	35	7	18	72	29	30	51
E	—	—	—	7	9	56	7	9	56

Abbreviations: NP=non-participants, P=participants; PS=performance status; ROD=rate of declining.

**Table 2:** Prediction of participation or declining to trials

	**Univariate analysis^a^**		**Multivariate analysis^b^**	
	**Odds ratio (95% CI)**	***P*-value**	**Odds ratio (95% CI)**	***P*-value**
Gender (male *vs* female)	1.008 (0.586–1.733)	0.977	0.646 (0.300–1.391)	0.264
Age (<60 *vs* ⩾60)	0.735 (0.432–1.250)	0.254	0.701 (0.376–1.310)	0.266
Smoking history (+ *vs* −)	1.394 (0.815–2.386)	0.225	2.538 (1.162–5.541)	0.019
Clinical stage (III *vs* IV)	0.608 (0.339–1.089)	0.093	0.681 (0.346–1.340)	0.266
PS (0 *vs* 1)	1.398 (0.792–2.467)	0.247	0.785 (0.396–1.554)	0.487
Physicians (A–E)		<0.001		<0.001

Abbreviations: NP=non-participant; P=participant; PS=performance status; ROD=rate of declining.

a By Pearson's *χ*^2^-test.

b By logistic regression analysis.

**Table 3:** Number of courses of the first-line chemotherapy

	**Clinical trial 1**		**Clinical trial 2**		
	**Participants**	**Non-participants**	**Participants**	**Non-participants**	***P*-value**
	100	16	96	57	
*First-line cycles*					
1	10 (10%)	4 (25%)	6 (12%)	4 (9%)	0.418^a^
2	18 (18%)	4 (25%)	8 (16%)	12 (27%)	
3	37 (37%)	7 (44%)	5 (10%)	9 (20%)	
⩾4	35 (35%)	1 (6%)	30 (61%)	20 (44%)	
					
*Gefitinib median duration (day)*			73	99	0.118^b^
Range			13–752	34–1065	
IQR			29–204	38.5–512	

Abbreviation: IQR=interquartile range.

a By Pearson's *χ*^2^-test.

b By log rank test.

**Table 4:** Treatment after the first-line chemotherapy

	**Participants**	**Non-participants**	
	**196 (%)**	**73 (%)**	***P*-value^a^**
*Chemotherapy regimen*			
0^b^	26	40	0.108
1	38	26	
2	22	25	
3	9	8	
>4	5	1	
			
Radiotherapy	49	34	0.031
Pleural or pericardial drainage	10	5	0.227
Operation on metastatic brain tumors	1	3	0.122
Early-phase trials	13	8	0.300

a By Pearson's *χ*^2^-test.

b Patients received first-line chemotherapy only.

**Table 5:** Clinical outcomes

	**Clinical trial 1**		**Clinical trial 2**		**Total**		
	**Participants**	**Non-participants**	**Participants**	**Non-participants**	**Participants**	**Non-participants**	***P*-value**
Response rate (%)^a^	29	12.5	32.3	40	30.6	34.2	0.569^b^
	(29/100)	(2/16)	(31/96)	(23/57)	(60/196)	(25/73)	
*Median follow-up time (day)*	329	339	493	444	388	406	0.846^c^
Range	45–2704	1–2176	36–2036	22–1688	36–2704	1–2176	
IQR	177–665	59–582	213–861	175–658	197–742	146–604	
							
*Median survival time (day)*	416	408	573	519	489	461	0.987^c^
Range	34–2704	53–2380	40–2036	35–1688	34–2704	35–2380	
IQR	264–815	140–698	251–938	276–1012	259–863	229–774	
1-year survival (%)	56.0	63.2	65.6	64.9	60.7	64.5	0.567^b^
2-year survival (%)	29.4	21.1	38.5	29.8	33.9	27.6	0.379^b^

Abbreviation: IQR=interquartile range.

a Excluding three patients who did not receive active treatment at our center.

b By Pearson's *χ*^2^-test.

c By log rank test.
